# Symplastic Leiomyoma in the Suprarenal Inferior Vena Cava

**DOI:** 10.5812/iranjradiol.10158

**Published:** 2012-12-27

**Authors:** Volkan Kahveci, Torel Ogur, Gokhan Cipe, Sevim Ozdemir, Selcuk Hazinedaroglu

**Affiliations:** 1 Department of Radiology, Etlik Training and Research Hospital, Ankara, Turkey; 2 Department of Surgery, Medical Faculty, University of Ankara, Ankara, Turkey

**Keywords:** Leiomyoma, Vena Cava, Inferior, Ultrasonography

## Abstract

Leiomyomas are benign tumors of the soft tissue and may develop in any location where smooth muscle is present. Leiomyoma in the inferior vena cava is a rarely seen pathology, and symplastic leiomyoma is also a rare histological variant of leiomyoma. In this case, we present a rare histological variant of symplastic leiomyoma in the inferior vena cava (IVC). This is the first radiologically reported case of a symplastic leiomyoma of the IVC.

## 1. Introduction

Leiomyomas are benign tumors of the soft tissue and may develop in any location where smooth muscle is present. They were first described by Virchow in 1854. These tumors are more frequently seen in peripheral blood vessels compared to the central vessels and have been identified in the vena cava in only a few cases (1). We present a case of symplastic leiomyoma, which is a rare variant of leiomyoma, together with MRI, ultrasonography (USG) and histopathological findings.

## 2. Case Presentation

**Figure 1:**
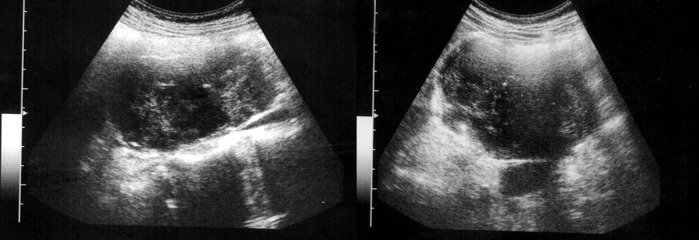
US imaging, heterogeneous echogenicity and lobulated contour mass

**Figure 2:**
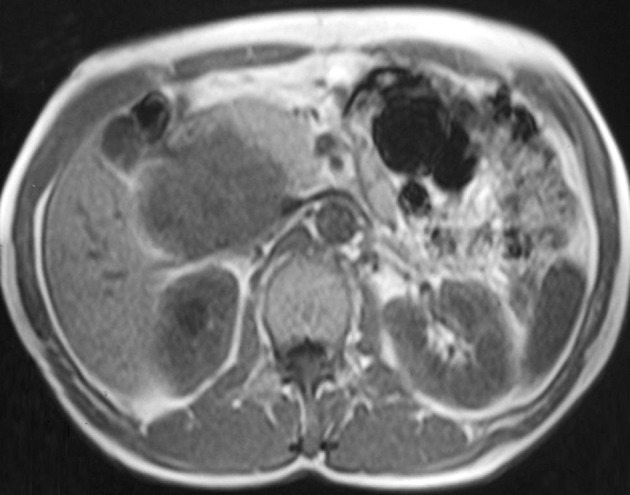
Axial T1-weighted MR image shows isointensity of the mass compared to the skeletal muscle.

**Figure 3:**
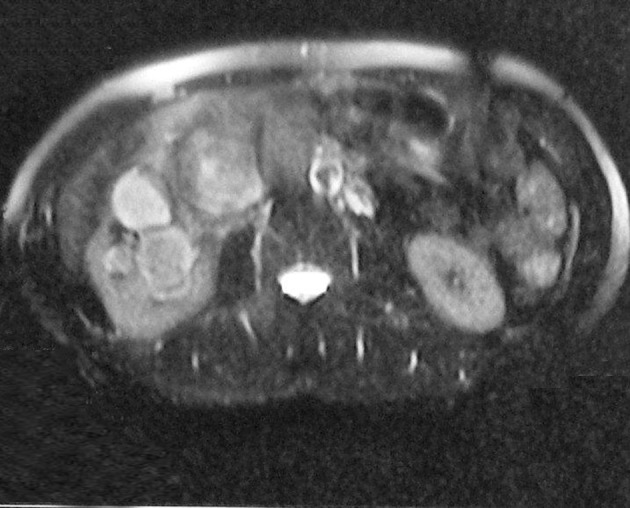
Fat suppressed axial T2-weighted MR image shows hyperintensity of the mass compared to the surrounding muscle tissue showing heterogeneous signal intensity.

**Figure 4:**
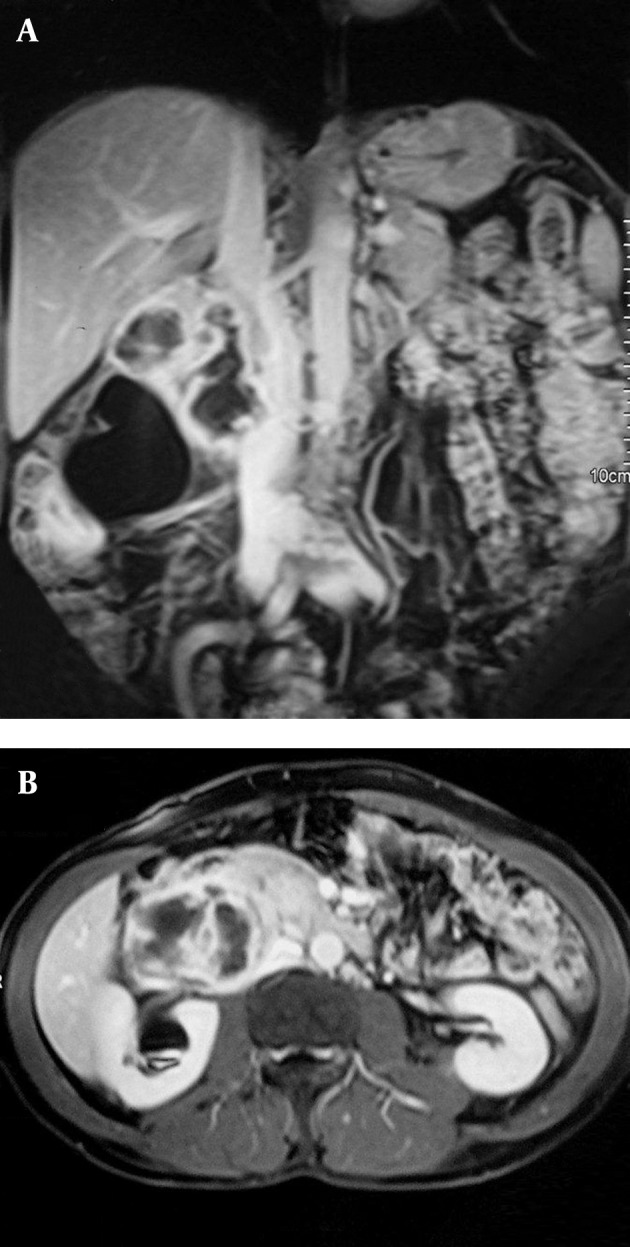
A. Coronal and B. Axial MRI following IV injection of contrast material shows a moderate level of heterogeneous contrast enhancement when compared to the surrounding muscle planes. The mass could not be separated from the inferior vena cava.

In the ultrasonography of a 54-year-old male patient with renal colic pain, we detected a renal stone and hydronephrosis and an incidental solid mass 88 × 67 × 100 mm in size right of the midline. It had heterogeneous echogenicity, lobulated contour and calcification foci, and was distinctly separated from the kidneys and liver, suggesting a pancreatic mass (Figure 1). In MRI, a retroperitoneal mass lesion was detected in the anteromedial adjacency of the upper-middle part of the right kidney. It had a calcification foci and was distinctly separated from the liver and kidneys, but not from the pancreas and inferior vena cava (IVC). It was isointense on T1-weighted images compared to the skeletal muscle (Figure 2) and hyperintense on T2-weighted fat suppressed sequences compared to the surrounding muscle tissue showing heterogeneous signal intensity (Figure 3). It was remarkable that the defined mass lesion gradually showed a moderate level of heterogeneous contrast enhancement when compared to surrounding muscle planes following IV injection of contrast material (Figure 4).

**Figure 5:**
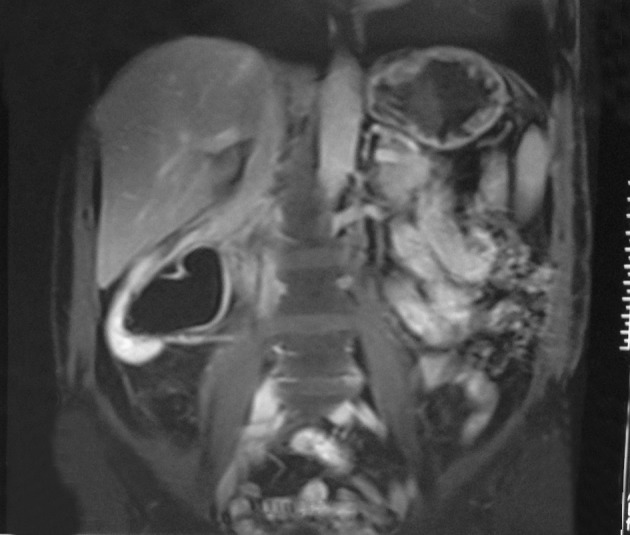
Coronal T1-weighted fat-suppressed contrast-enhanced MR image of the patient one month after surgery. There is no residual mass around the inferior vena cava.

The retroperitoneal mass was excised and pathologically examined. It was an encapsulated, cream-colored 10 × 6 × 4cm sized mass. An immunohistochemical examination showed positive results of smooth muscle actin (SMA) staining and negative results with p53 and S-100. The Ki-67 proliferation index was up to 10%. Accordingly, the histopathological diagnosis was considered as symplastic leiomyoma. One month after operation, MR imaging was performed and no residual mass was determined around the IVC (Figure 5). IVC was patent and healing in the hydronephrosis of the right kidney was noticed.

## 3. Discussion

The role of MRI in the evaluation of retroperitoneal soft tissue masses is becoming more important. Localization of the lesion, pattern of spread, specific components and vascularity of the tumor narrow the spectrum of the differential diagnosis (2, 3).

In our case, the mass had a right sided retroperitoneal localization which seemed to originate from the IVC and pancreas, because it could not be distinctly separated from them. However, the chance of a potential diagnosis of a pancreatic mass lesion is decreasing due to absence of laboratory and clinical findings to support pre-diagnosis and imaging evidence, in addition to MR imaging findings. Primary smooth muscle tumors originating from vessels are quite rare and most of them are malignant. Leiomyosarcomas are the most common primary malignancy of IVC that generate more than 90% of all reported IVC sarcomas. There are intimal and mural subtypes of IVC sarcomas according to their origin. Intimal ones, which are also called luminal, are differentiated sarcomas, undifferentiated pleomorphic sarcoma and angiosarcoma. Mural sarcomas are usually thought to develop into leiomyosarcomas from the muscle of media. Leiomyomas also arise from the smooth muscles of media (4-6). Since there was no detection of pathological signal changes consistent with myxoid stroma in mass lesions of our patient, a diagnosis of malignant fibrous histiocytoma was excluded.

In our case, the delayed pattern of enhancement was present. This pattern is often seen in neurogenic tumor subtypes of benign tumors and in a few malignant tumors, such as desmoid tumors, hemangioma, leiomyoma and also myxoid liposarcoma, leiomyosarcoma and malignant lymphoma (2, 3). An enhancement pattern can vary depending on the type of leiomyoma. While cellular leiomyomas show contrast enhancement in the early phase of dynamic examination, degenerated leiomyomas can show delayed and irregular contrast enhancement (7).

Leiomyomas and leiomyosarcomas are round or lobulated masses with regular contours. MR images of the lesions depend on the amount of fibrous and smooth muscle cells in each lesion. Lesions are similar to skeletal muscle on T1-weighted sequences; however, most of them show heterogeneous signal intensity. Typical appearances are hypo- or isointense on T1-weighted images and hypointense on T2 weighted images. Solid components often show contrast enhancement following injection of contrast material intravenously. Non-degenerated uterine leiomyomas have a typical appearance on MR images. They are well-defined masses that show hypointense homogeneous signal intensity on T2-weighted images compared to the muscle tissue. Cellular leiomyomas show relatively higher signal intensity on T2-weighted series and contrast enhancement following injection of contrast material intravenously (7). Similar to cellular leiomyomas, vascular leiomyomas also show hyperintense signal intensity on T2-weigthed series and contrast enhancement (8, 9). Degenerated leiomyomas have variable appearances on T2-weighted images and contrast-enhanced examination. Calcific degenerated leiomyomas have low signal intensity on T2-weighted series and their appearance is similar to that of standard leiomyomas (7). Hyalinization within the mass is hypointense in all sequences and shows minimal central heterogeneous contrast enhancement (9). Leiomyomas with cystic degeneration have higher signal intensity on T2-weighted series and those areas do not show contrast enhancement. Leiomyomas with myxoid degeneration have very high signal intensity on T2-weighted series and minimal contrast enhancement is remarkable on the contrast-enhanced images of those areas. In our case, similar to typical leiomyomas, iso-intense signal intensity was detected with the muscle cells on T1-weighted series, but unlike typical leiomyomas, it had hyperintense signal intensity on T2-weighted sequences similar to that of cellular type leiomyoma and vascular leiomyoma. The tumor originates from the tunica media of the IVC and contains a large number of vessels which may be the reason of significant contrast enhancement (10).

Histopathologically, there are typical, epithelioid, cellular, hemorrhagic cellular, lipoleiomyoma and symplastic variants of leiomyoma (11). The term "bizarre" was first described for gastric leiomyoma by Martin and colleagues in 1960. Then, it was adopted as "bizarre leiomyoma" by the World Health Organization (WHO) and described as "leiomyoma containing giant cells without mitotic activity or with small and pleomorphic nuclei". The synonyms of this term have been reported as "atypical", "pleomorphic" and "symplastic" (12). The symplastic leiomyoma with a mitotic index lower than 10 mf/10 is a smooth muscle tumor which is rarely reported in the medical literature (13, 14). Only one case of IVC involvement was reported (15).

Growth patterns of leiomyoma variants are able to mimic that of more aggressive cancers. Disseminated peritoneal carcinomatosis may resemble peritoneal leiomyomatosis; parasitic leiomyoma or intravenous leiomyomatosis may resemble leiomyosarcomas; intravenous leiomyomatosis may resemble renal cell carcinoma in the involvement of IVC; and benign metastatic leiomyoma may resemble pulmonary or hepatic metastatic disease (16).

Pleomorphic tumor cells with distinct atypical nuclei and low mitotic rate are important characteristics of the variants of the symplastic leiomyoma. Most pleomorphic cells often have multinucleated or multilobule nucleus, but the large and dilated mononuclear cells can be observed. These multinucleated cells can be present as focal, multifocal or diffuse throughout the mass, and generate more than 25% of the tumor in most cases. Although these tumors usually have degeneration, edema and hyaline changes, there is no coagulative tumor cell necrosis. Symplastic cells are observed more frequently on the edges of degenerative areas (11). It may be misdiagnosed as leiomyosarcoma due to the marked nuclear atypia. Mitotic activity is usually a commonly used criterion to make the distinction between atypical leiomyoma and leiomyosarcoma. The mitotic index of leiomyosarcoma is equal to or more than 10 mf/10 hpf. In the literature, it has been reported that symplastic leiomyoma with high cellularity has a benign course and the mitotic activity of atypical cells is 2-7mf/10hpf (11, 13).

In this study, we attempted to present a rare histological variant of vascular leiomyoma. It is difficult to identify the origin of these large tumors with imaging studies. As with other defined vascular leiomyomas, MRI shows signal intensity depending on the histopathological changes and tissue content. This rare variant can easily be confused with leiomyosarcoma (15). Examinations using MRI are quite valuable in the evaluation of extensions and in planning surgery.
